# Doctors and nurses benefit from interprofessional online education in dermatology

**DOI:** 10.1186/1472-6920-11-84

**Published:** 2011-10-14

**Authors:** Thomas Schopf, Vibeke Flytkjær

**Affiliations:** 1 Norwegian Centre for Integrated Care and Telemedicine, University Hospital of North-Norway, P.O. Box 6060, 9038 Tromsø, Norway

## Abstract

**Background:**

Benefits of online learning in the health sector have been demonstrated in previous studies. We examined the potential benefits of a joint web-based curriculum on atopic eczema for health personnel.

**Methods:**

Enrolled doctors and nurses had access to the curriculum for 8 weeks. After the course learners completed a questionnaire. Two dermatologists rated the quality of the submitted homework assignments. Based on data from the project's budget and the Norwegian Medical Association, we estimated the saved travel expenses.

**Results:**

Eighty-eight learners (46 doctors) registered for the course. We received 55 questionnaires (response rate 63%). Twenty-seven learners (31%; 16 doctors, 11 nurses; χ^2 ^= 0.03; P = 0.87) used the discussion forum. We found no significant differences in the total questionnaire scores between doctors and nurses. The homework assignments were given an average score of 3.6 for doctors and 3.5 for nurses (P = 0.8) by rater 1. Rater 2 scored 3.9 and 3.6 for doctors and nurses respectively (P = 0.2). The break-even between travel/hotel expenses and course development costs occurred at 135 saved travel refund applications.

**Conclusions:**

Doctors and nurses were equally satisfied with a joint web-based course on atopic eczema. The use of an online discussion forum was limited but similar between doctors and nurses. There were no significant differences in the quality of submitted homework assignments. The cost of developing the course was 716 841 NOK and the first 86 learners saved 455 198 NOK in travel expenses.

## Background

Atopic eczema (AE) is a common chronic skin disease in children and has a considerable impact on the quality of life of the entire family [1]. The key role of nurses in the management of AE patients has been demonstrated [2-4]. In the nurse-led consultation model nurses are responsible for teaching patients basic treatment techniques while doctors mainly focus on diagnosis and selection of treatment. Because of the high prevalence of AE in Northern Europe and the concomitant lack of specialists, only a minority of patients will see a dermatologist or paediatrician [5]. As a consequence, most AE patients consult general practitioners (GPs) and/or community nurses when seeking help for their skin complaints. Patients inevitably will compare the advice given by nurses with that given by doctors. Providing consistent advice seems important in order to avoid confusion [2]. A clear definition of each profession's responsibility and role appears essential.

The continuing medical education (CME) programme for GPs in Norway includes several dermatology courses but there is no comprehensive curriculum on AE dealing with basic skin care and the various treatment options in detail [6]. The access to CME for community nurses in Norway seems limited. In a survey, 68% of community nurses said they knew about courses they needed but could not attend because of practical difficulties [7]. While doctors and nurses in Norway traditionally have attended separate CME programmes specific to their profession, there has been increased interest in continuing interprofessional education elsewhere [8,9].

Online education has emerged as an alternative to ordinary classroom lessons and does not require participants and teachers to be physically present at a specific site and time [10]. Consequently, the need for travelling may be reduced. GPs attending CME in Norway regularly obtain refunding of travel and hotel costs from the Norwegian Medical Association (NMA) whereas nurses usually are refunded by their employers [11].

In 2008 we developed "Help, it's itchy!", a web-based course aimed specifically at doctors and nurses. The idea was to educate doctors and nurses in the management of AE in general practice including basic skin care techniques. Two board certified dermatologists and a board certified paediatrician wrote the content of the curriculum. An experienced GP, 3 consultant dermatologists and the author VF who is a specialist nurse, reviewed the course. Prior to this study the educational boards of family medicine of the NMA as well as the Norwegian Nurses Organisation (NNO) reviewed and approved the course for CME credits for doctors and nurses.

We designed the course on the basis of social constructivist learning theory and experiential learning principles [12,13]. The intended pedagogical approach was mainly through presentations of clinical cases showing typical problems seen in general practice. In order to break down the complex clinical scenarios into simpler lessons, the curriculum was organised in 3 modules: 1. Steroids and calcineurin inhibitors, 2. Infections and 3. Allergies. Although learners through the course menu could decide which sections to study first, in the default setting the complexity of the learning material (both within and between sections) increased gradually.

In a threaded discussion forum learners were encouraged to submit questions and share their own experiences. Questions were answered by the facilitator (author TS) and/or commented by other learners. At start-up the facilitator asked learners to introduce themselves in a social thread. Incoming requests and their answers were organised in threads according to the topic. We systematically added comments and similar questions received later to those threads. All communication was asynchronous.

Course content was presented as narrative text and in audiovisual format. While some subsections were marked "especially recommended for doctors or nurses", all learners were encouraged to study these sections. Clinical examples were presented with photographs showing various stages of AE. Video sequences and photo series were used to demonstrate various therapeutic techniques: applying emollients, preparation of facial dressings, preparation of wet-wraps and preparation of potassium permanganate baths. Various information sheets were shown to exemplify the instruction of patients. Links to other online resources for patients as well as health personnel were presented. In every module there was an optional test set with 8 - 9 multiple choice questions for self-assessment. After submission of all answers in a particular test set, learners automatically received feedback. To complete the course, every learner had to submit a homework assignment involving 3 hypothetical patient cases illustrated with photographs (see additional file 1). A total of 9 questions about treatment recommendations had to be answered. The homework patient cases including all questions were accessible at any time during the course. The facilitator provided learners with feedback including a copy of the submitted answers 3-5 days after submission.

We used the online learning management system Helsekompetanse.no from the Norwegian Centre of Integrated Care and Telemedicine (NST) to design the course [14]. Helsekompetanse.no is based on open source and there is no licence required to use the platform.

The aim of the study was to answer these questions: Are doctors and nurses equally satisfied with a web-based course on the management of AE in general practice? Is there any difference between doctors and nurses in the use of an online discussion forum? Is there any difference between doctors and nurses in how virtual patients are managed in the homework assignments? How much can be saved in terms of reduced travel costs?

Our hypothesis was that doctors and nurses would perceive the course equally and that their frequency of use of the discussion forum would be equal. We anticipated a higher quality level in the homework assignments submitted by doctors. We expected considerable travel savings in the long term.

## Methods

We report on the perceptions of physicians and nurses after participating in a web-based course on AE. The course was held twice in 2009 for two different mixed groups of learners. Each course offered unlimited access to the entire curriculum for 8 weeks. After initially receiving an individual user name and password, course participants were free to access the curriculum at any time and place in this period. At the end of the course, learners were asked to complete an anonymous online questionnaire. We examined satisfaction with the course including: Presentation of content, medical relevance of content, instructional design, interactivity and ease of use. Learners were asked to rate their agreement on 15 statements by the use of a Likert scale in addition to 6 open-ended qualitative questions. The questionnaire was adapted from similar questionnaires used previously for the evaluation of various online health courses designed at the NST. Use of the curriculum was established through computerised logging. The number of participants' postings in the discussion forum was counted manually. Postings in the discussion forum were classified as educational or social. Administrative messages (e.g. about practical issues regarding CME credits), "Thank you"s and postings from the facilitator were not counted.

A random selection of 60 answers to the homework assignments (30 answers each from doctors and nurses respectively) submitted by course participants were rated in a blinded manner by two independent board certified dermatologists not involved in the project. The quality of the answers was rated from 1-5 (1 = very poor; 2 = poor; 3 medium; 4 = good; 5 = very good). The 9 questions answered by a particular learner were identical for doctors and nurses. (A tenth question was not analysed in the study because it was intended for doctors only).

We assumed that participation in our online course replaced a traditional course. Based on data from the NMA on real refund applications we calculated the hypothetical saved travel and hotel accommodation expenses. Refund data for 2007 were analysed [15]. The costs for developing the course were estimated through logging of working hours of involved staff excluding research work.

The course was advertised on the websites of the NMA and the NNO. Physicians and nurses from all over Norway could register. Assuming equal groups and a drop-out rate of 40%, 80 participants would be required to show a difference between doctors and nurses in the total questionnaire score of 5 points with a power of 80% and significance level of 5% (SD = 6). Except for professional background, login times, homework submissions and costs, data were collected through online questionnaires. The maximum possible total score in the questionnaire was 75, indicating a high level of satisfaction, while the minimum possible score was 15. We used student's t-test to compare the mean total questionnaire scores and the mean homework scores between doctors and nurses. Questionnaire scores from single questions were compared using the Mann-Whitney U-test. The percentages of submitted homework assignments and discussion postings were compared by Chi-Squared statistic. Interrater agreement between the scoring dermatologists was determined by intraclass correlation. Responses to the open-ended questions were analysed by identifying common themes and grouped accordingly. All data analyses were performed using the PASW 18 programme (SPSS Inc., Chicago, USA).

This study does not report experimental biomedical research and did not require approval by the Regional Committee for Medical and Health Research Ethics in Northern Norway. All participants gave informed consent.

## Results

A total of 88 persons registered to participate. We received a total of 55 questionnaires (response rate 63%; 31 doctors, 19 nurses, 2 students, 3 unspecified). Sixty-four percent of learners had a working experience of more than 5 years. See table 1 for data on the professional background of participants.

**Table 1:** Background of learners based on registration data

Learner	General practice	Hospital	Other	All
Doctors	43 (49%)	3 (3%)	-	46 (52%)

Nurses	16 (18%)	20 (23%)	4 (5%)	40 (45%)

Students	-	-	2 (2%)	2 (2%)

The mean total login time per learner was 271 minutes (doctors: 295 minutes; nurses: 242 minutes; N = 86; P = 0.46). Eight learners (9%; 5 doctors, 3 nurses) never logged in, while 8 learners (9%; 1 doctor, 7 nurses) had a login time of less than 10 minutes during the entire course period. Twenty-seven learners (31%; 16 doctors, 11 nurses; χ^2 ^= 0.03; P = 0.87) made a total of 53 postings in the discussion forum. Forty-seven percent of postings were of social character (doctors 50%; nurses 44%), the remainder was educational.

### The questionnaires

The mean total questionnaire score was 64.5 (doctors: 64.7; nurses: 64.5; P = 0.95). The analysis of single questions showed no significant differences except in one item. Doctors scored significantly higher on the statement "I would like to get access to the curriculum after the course has finished."; doctors: median = 5; nurses: median = 4 (P = 0.045). See table 2 and 3 for more information. Concerning the open question about mixing of professions, 15 learners (6 doctors, 9 nurses) commented that a mix of professions in the course was positive. Six learners commented neutral (4 doctors, 2 nurses). No negative comments were identified on this question. See table 4 for details on the open-ended questions.

**Table 2:** The 5 items with the highest scores

Item	Mean score: 1 = strongly disagree; 5 = strongly agree	
	**All**	**Doctors/nurses**

I did not need help to use the software.^1^	4.9	4.9/4.9

The course was useful to me and my work.	4.8	4.9/4.7

The content of module 1 was relevant.	4.8	4.8/4.8

The exams were useful.	4.8	4.8/4.7

The content of module 2 was relevant.	4.7	4.7/4.8

^1 ^Item has been reversed.

**Table 3:** The 5 items with the lowest scores

Item	Mean score: 1 = strongly disagree; 5 = strongly agree	
	**All**	**Doctors/nurses**

I have participated actively in the discussion forum.	1.9	1.8/1.8

The option to exchange experiences in the forum has been valuable.	2.9	2.9/3.1

The option to ask questions in the forum has been valuable.	3.7	3.6/3.7

The instruction videos have been useful.	4.4	4.4/4.3

I would possibly use some sections of the curriculum for the instruction of patients.	4.4	4.5/4.2

**Table 4:** The 10 most common themes identified in the open questions

Common themes from open questions	Number of comments	
	**All**	**Doctors/nurses**

"A great course."	32	21/11

"Great flexibility when to login."	10	7/3

"The discussion forum was valuable"	9	7/2

"The software was easy to use."	8	5/3

"I didn't have enough time to use the discussion forum."	7	5/2

"I'd like more clinical cases."	6	5/1

"I'd like to do a similar course on other topics."	5	5/0

"Demonstrations by video were useful."	4	2/2

"I read the postings in the discussion forum without actively contributing."	4	3/1

"I had problems using audio/video files."	4	3/1

### The homework assignments

Fifty-nine learners (67%) completed the course by submitting a total of 177 answers (3 answers per learner) in the homework assignments. While 74% of the doctors made a submission, 58% of the nurses did so (χ^2 ^= 2.9; P = 0.09). The answers were given an average score of 3.6 for doctors and 3.5 for nurses (P = 0.8) by rater 1, while rater 2 scored 3.9 and 3.6 for doctors and nurses respectively (P = 0.2). The intraclass correlation coefficient (Yates correction average measures) was 0.7. See table 5 for more details.

**Table 5:** Mean scores of homework assignments

Part of homework	Rater 1 Doctors	Rater 2 Doctors	Rater 1 Nurses	Rater 2 Nurses
Case 1	3.5	4.2	3.6 (P^1 ^= 0.5)	3.6 (P^1 ^= 0.06)

Case 2	3.9	4.0	3.6 (P^1 ^= 0.4)	4.0 (P^1 ^= 1)

Case 3	3.1	3.4	3.1 (P^1 ^= 1)	3.3 (P^1 ^= 0.7)

All	3.6	3.9	3.5	3.6

^1^P-values relate to differences by profession

### Travel savings

The average refund for educational travel/hotel expenses was 5 293 NOK per GP and application (total number of applications received: 4 610). The participants of our course potentially saved 455 198 NOK (5 293 NOK × 86 learners) in travel/hotel costs.

The expenses for developing the online course were estimated to be 716 841 NOK. Break-even between travel/hotel expenses and course development costs was found at 135 saved refund applications (716 841 NOK: 5 293 NOK).

## Discussion

For the first time in Norway a web-based CME course in dermatology designed for doctors and nurses was implemented. Based on our findings, participating doctors and nurses seemed to be equally satisfied with the curriculum. We found no significant differences in the total questionnaire scores. A difference was found only in one item: doctors seemed to be more interested in getting access to the curriculum after the course had ended. One might speculate if doctors are more familiar with the use of references in their daily work compared to nurses. Nevertheless, both doctors and nurses said they would like to use parts of the curriculum for the instruction of patients. Doctors and nurses scored equally on both relevance of content and usefulness for daily work.

The percentage of learners completing the course was higher among doctors. Doctors also performed slightly better in the management of the patient cases in the homework assignments, but these differences were not significant. We assume that a possible explanation for the higher completion rate could be motivation. In Norway GPs are obliged to obtain a certain number of CME credits in order to be recertified every five years. In contrast, nurses are not obliged to complete CME courses in the same way.

Learners could interact with the facilitator or with other learners in the discussion forum. We had hoped for more online discussions. However, both doctors and nurses appreciated reading threads in the discussion forum even if they did not post anything. "Lack of time" was a typical explanation given for the limited use. Time constraints as a barrier to interaction in online education have been reported before [16].

Web-based courses can easily be reused. Also updating learning content according to new developments in medicine is easy to accomplish. The use of license-free open source software may keep course expenditure low [17]. In Norway the NMA funds the administration of most web-based CME courses. In contrast, time and costs for the creation of distance learning are rarely compensated [18]. Refund of travel and accommodation costs for doctors attending traditional courses is an additional burden for the NMA [15]. Savings due to reduced travel may instead be used to cover some of the high costs involved in the creation of distance learning. Theoretically the potential travel savings associated with the 2 online courses in our study equaled more than half of the developing costs. While the ability of online education to reduce travel expenses obviously has been acknowledged by private companies [19], there seem to be few reports in the medical literature investigating this issue [20-22]. In a review Brown and co-workers report that economic evaluations of CME are rare [23]. In a study by Walsh e-learning appeared more cost-effective compared to traditional learning methods for GPs [24]. Our results suggest that savings due to reduced travel expenses should be taken into account when online CME programmes are planned.

### Strength and limitations

A strength of our study was that the course was advertised as part of the regular CME programmes for doctors and nurses. This probably minimised bias in the selection of participants. We note the following limitations: First, based on the reported data on homework performance, we cannot conclude on a change of learning outcomes as baseline data are missing. Nevertheless, the results of the homework assignments suggest that doctors and nurses have similar knowledge levels in the management of AE patients. Second, we do not know if the curriculum contributed to increased collaboration between doctors and nurses. Third, in our estimation of saved travel expenses we assumed that online education would replace some of the traditional CME courses, although we do not know how realistic this is. Many learners may still wish to travel to a traditional course and possibly consider the online course as a supplement only. Fourth, we have not conducted a full economic analysis. Our data merely suggest that saved travel expenses should be included in future analyses of cost effectiveness in distance education.

## Conclusions

Continuing interprofessional education in dermatology for GPs and nurses appears beneficial. A joint web-based course on AE in general practice was equally appreciated and seems sustainable over time. Learners perceived time constraints a barrier to interaction. There is a potential of saved travel expenses already in a short term perspective.

## Competing interests

The authors declare that they have no competing interests.

## Authors' contributions

TS had primary responsibility for protocol development, data analysis and writing of the manuscript. VF contributed to data collection, data analysis and writing of the manuscript. All authors read and approved the final manuscript.

## Pre-publication history

The pre-publication history for this paper can be accessed here:

http://www.biomedcentral.com/1472-6920/11/84/prepub

## Supplementary Material

Additional file 1 **Homework assignment questions (clinical images in case 1 and 2, not shown)**. Click here for file
