# Supplementary material for: Doctors and nurses benefit from interprofessional online education in dermatology
Source: BMC Med Educ. 2011 Oct 14;11:84. doi: 10.1186/1472-6920-11-84 (PMC3212800; doi:10.1186/1472-6920-11-84)
Supplement: Additional file 1 — Homework assignment questions (clinical images in case 1 and 2, not shown). [file 1472-6920-11-84-S1.DOC]

Additional file 1 - Homework assignment questions (clinical images in case 1 and 2, not shown)

Case 1

The patient is a 7 year old boy whose eczema flared in the last 10 days. He has been treated with over-the-counter topical steroids for 5 days without any improvement.

Which type of steroid do you intend to use on the trunk and upper extremities? Describe a treatment regimen including a tapering plan.

There is also eczema on the genitals and in the groins. Which kind of steroid would you use for this?

When is it appropriate to see the patient for a clinical follow-up? If the treatment has failed then, what do you do next?

Is tacrolimus an alternative treatment? Consider both the first consultation and the follow-up appointment.

Case 2

The patient is a 4 year old girl who was diagnosed with atopic eczema during her first year of life. She has suffered during the last 6 months with itch and skin eruptions. Since two weeks there has been a flare affecting the entire body including the face. Her mother has treated her with topical steroids. On the trunk and extremities she used betamethasone valerate, but does not recall the name of the drug used on the facial skin. The condition has improved on the trunk, but on the face and the upper extremities it is virtually unchanged.

Describe the skin findings on the facial skin and hands. Consider if there are signs of infection.

Which additional information would you ask the patient?

Set up a treatment regimen for the face and the upper extremities.

Case 3

Anne, 5 years old, is seen by the doctor together with her parents. She was diagnosed with atopic eczema and asthma 2 years old. The eczema has always been worse after the ingestion of eggs. The last 6 months her mother has noticed a possible rash after ingesting fish and citrus fruits. She is uncertain whether Anne has a food allergy.

Describe the typical scenario of a fish allergy.

How would you investigate Anne? Consider if testing is indicated and if so, which tests should be done?

The following question is for doctors only: Specific IgE for fish shows 40 kU/l. How do you interprete this? What advice do you give to the mother?
